# Role of Perceived Competence and Task Interest in Learning From Negative Feedback

**DOI:** 10.3389/fpsyg.2022.830462

**Published:** 2022-02-18

**Authors:** Dajung Daine Shin, Sung-il Kim, Myung-Jin Lee, Yi Jiang, Mimi Bong

**Affiliations:** ^1^Department of Education, Brain and Motivation Research Institute (bMRI), Korea University, Seoul, South Korea; ^2^Division of Educational Evaluation, Korea Institute for Curriculum and Evaluation, Jincheon-gun, South Korea; ^3^Department of Educational Psychology, Faculty of Education, East China Normal University, Shanghai, China

**Keywords:** task interest, perceived competence, negative feedback, cognitive control, learning, feedback processing

## Abstract

We used functional magnetic resonance imaging to examine the interactive effects of perceived competence and task interest on the cognitive and affective responses to negative feedback. Twenty-four undergraduates performed both interesting and uninteresting tasks and received failure feedback. The participants’ perceived competence in the task was manipulated between subjects prior to scanning with bogus feedback. The results showed that negative feedback processing was contingent upon both perceived competence and task interest. The most adaptive coping mechanism, indicated by activation in the cognitive control network and attenuation in the negative affect region, was identified for the high-competence and high-interest combination. When either competence or interest was low, signals in the cognitive control network were weaker. The most detrimental activation patterns were observed for the combination of low-competence and high interest. Our results reveal the combination of task and learner characteristics that best harnesses the potential benefits of negative feedback and illustrate the neuroscientific mechanisms underlying this observation.

## Introduction

Feedback lies at the heart of learning and goal attainment. It provides learners with information about the desired goal state and thus motivates them to optimize their behaviors to reach that goal. Many classical theories on learning posit that positive feedback leads to a positive affect and fosters motivation, while negative feedback generates a negative affect and undermines motivation (e.g., Atkinson, 1964). While this assumption generally holds, several factors are known to moderate the relationship, particularly in relation to negative feedback. This is because, unlike positive feedback, which is invariably rewarding and informative, negative feedback can be construed as either punishing or informative depending on the context and the person (Audia and Locke, 2003; Lempert and Tricomi, 2016; Kim et al., 2018). For example, negative feedback could be especially threatening to students who lack competence in performing a task relative to those who are highly competent. Students could also feel greater distress when they fail in a task that they value highly. Despite this variability, the moderating roles of individual and contextual factors on negative feedback processing and their underlying mechanisms remain elusive. The present study sought to address this issue by examining the differential cognitive and affective reactions to negative feedback according to perceived competence and task interest using brain imaging.

Successful coping with and learning from negative feedback involves two essential processes: cognitive control and emotion regulation. As with any type of feedback, negative feedback points to a gap between the learners’ goal and their current state. Because failure indicates that the present performance has not reached the desired level, negative feedback additionally advises them to adjust their behaviors and strategies. To benefit from negative feedback, therefore, individuals need to diligently monitor errors and modify their actions to improve subsequent performance (Holroyd and Coles, 2002). Cognitive control is the set of executive processes responsible for these goal-directed actions, encompassing performance monitoring, error detection, response inhibition, action selection, and decision making (Cole and Schneider, 2007). In the brain, activity in the insula, anterior cingulate cortex (ACC), inferior prefrontal cortex (PFC), dorsal striatum, and dorsolateral prefrontal cortex (dlPFC) underlie these cognitive control processes (Ridderinkhof et al., 2004; Cole and Schneider, 2007; Niendam et al., 2012). These cortical regions interact and work in a complementary manner to allow intricate cognitive control in response to failure (Ullsperger et al., 2014; Woo et al., 2015).

At the same time, learners must suppress negative emotions provoked by negative feedback, which could easily interfere with their ability to exercise cognitive control. Negative feedback is inherently discouraging and thus generates an aversive affect, such as frustration, anger, and anxiety. Activity in the thalamus and amygdala, in which signals of unpleasant stimuli travel from the former to the latter, is known to represent the experience of negative emotions (LeDoux, 1994; Das et al., 2005; Choi et al., 2012). The inability to overcome negative emotions can result in unsuccessful or maladaptive coping with failure (for a review, see Gross, 2015 and Skinner and Saxton, 2019). Prior meta-analyses have identified a set of common cortical regions that enables the regulation of negative emotion or the downregulation of amygdala activity, including the bilateral temporal gyrus, angular gyrus, and ventromedial prefrontal cortex (vmPFC; Buhle et al., 2014; Kohn et al., 2014). The recruitment of these brain regions was reported in the employment of diverse strategies to regulate negative emotion, such as reappraisal and suppression (e.g., Goldin et al., 2008).

A setback can be appraised differently depending on the characteristics of the individual and/or the context, thus leading to different cognitive and emotional responses to negative feedback. A learner’s perceived competence in a given task can be a vital moderating factor. Individuals with low self-esteem or self-efficacy are more likely to feel threatened when receiving negative feedback and try to avoid it (Kluger and DeNisi, 1996). Conversely, highly self-efficacious individuals tend to view negative feedback as valuable information for improving their performance and are likely to persevere (Audia and Locke, 2003; Lee et al., 2020). These perceptions may, in turn, facilitate the cognitive control and emotion regulation of highly competent individuals when faced with negative feedback and impede those of low-competence individuals. Indeed, Kim et al. (2010) found that students with low competence exhibited greater activation in the brain regions involved in aversive emotions when provided with norm-referenced feedback than did those with high competence.

Task interest is another important factor that can moderate the process of negative feedback. It is widely accepted that feeling interest is advantageous for motivation and effort in taxing achievement situations (Ainley et al., 2002; Schweinle et al., 2006). When learners engage in an interesting task, they are prone to believe that their engagement is guided by enjoyment and persist in the face of challenges (Deci and Ryan, 1987; Krapp, 2002; Hidi and Renninger, 2006). For instance, Kim et al. (2008) found that task interest operates as an effective buffer against the detrimental effects of negative performance feedback. Students in the low-interest condition reported stronger avoidance goals after receiving failure feedback on the task. Those in the high-interest condition, in contrast, did not report higher avoidance goals even after receiving failure feedback. Fulmer and Frijters (2011) also observed that, when given an excessively challenging reading task, students who read a text that was personally interesting to them were twice as likely to persist in the task and made more adaptive attribution for the difficulty with the task. Although there is a paucity of direct neuroscientific evidence, the buffering effect of interest in the face of adversity may be underpinned by the stronger execution of cognitive control and emotion regulation.

While perceived competence and task interest may independently play a moderating role in negative feedback processing, they are likely to interact with each other and jointly influence the process. Recent studies on the expectancy-value theory (EVT) offer direct substantiation of this. The modern EVT posits that, if individuals do not feel efficacious in a task that they perceive high value (i.e., a task that they think is useful, important, interesting, or low-cost), this could lead to various negative outcomes, including poor performance (Trautwein et al., 2012), anxiety (Nie et al., 2011), and the frequent use of defensive strategies (e.g., cheating and procrastination; Lee et al., 2014). Subsequent experimental studies have reported that high competence is an essential learner characteristic that is required to benefit from the perception of high value (Canning and Harackiewicz, 2015; Durik et al., 2015). Therefore, interest has a synergetic effect only among individuals with high competence, while it amplifies, rather than buffers, negative consequences among individuals with low competence. Researchers postulated that threatened self-views may be responsible in the latter case (Eccles and Wigfield, 1995; Lee et al., 2014). That is, perceiving high value in a task in which individuals are not competent can damage their self-worth and increase their anxiety (Covington, 2009).

Despite this sound evidence, it is unclear whether interactive patterns between competence and interest operate when an evaluative threat is encountered. The present study thus utilized functional resonance imaging (fMRI) to investigate the processes that occur in a learner’s mind when failure feedback is received. In doing so, we sought to determine the biological underpinnings for different mechanisms for coping with failure *via* competence and interest. Using fMRI is especially useful for the purpose of the present study because it allows objective access to participants’ cognitive and affective processes at the exact moment that they receive negative feedback, which is difficult to achieve with a survey or behavioral observations (Panksepp, 2005; Kim et al., 2010). We used a 2 (between-subject factor: high-competence vs. low-competence) × 2 (within-subject factor: interesting task vs. uninteresting task) mixed design. The participants’ neural responses after receiving bogus failure feedback were analyzed by group and task.

We hypothesized that an interesting task would be especially helpful for the high-competence group in coping with negative feedback. That is, positive motivation engendered by interesting tasks was expected to play a synergetic effect with high competence in overcoming the detrimental cognitive and emotional reactions triggered by negative feedback. This synergy would be evident through the greater recruitments of the emotion regulation and cognitive control systems. On the contrary, we expected that task interest would hinder the low-competence group from grappling with the negative feedback. This would be associated with reduced activity in the cognitive control system in the low-competence group during negative feedback processing for the interesting task. We also predicted the less attenuated regional activity in the amygdala and thalamus for the low-competence and high-interest condition, further reflecting the impeded regulation of negative emotion. The results of this study would provide greater insights into when and for whom negative feedback functions as a learning opportunity.

## Materials and Methods

### Participants

Thirty healthy right-handed undergraduates (15 males; mean age = 21.4) participated for a monetary reward. All participants were prescreened for the absence of any psychiatric and neurological conditions and provided informed consent. The procedures in the study were approved by the university’s Institutional Review Board. The participants were randomly assigned to either the high-competence (*n* = 17) or low-competence (*n* = 13) group. Five participants were excluded from the analysis due to excessive head motion (>3 mm) during the fMRI experiment. The final sample consisted of 13 students (seven males) in the high-competence group and 12 (six males) in the low-competence group.

### Task and Procedures

We used a perceptual judgment task (Kim et al., 2008) in which participants were asked to evaluate whether the number of target stimuli was 15 or not as quickly as possible. We chose this task because it was deemed sufficiently novel to most college students. Using a novel task was necessary to enable both competence manipulation and bogus performance feedback. There were two within-subject task conditions that differed according to how interesting the target stimuli were. The interesting task condition involved the faces of famous comedians in Korea. The comedians’ faces with funny expressions and comical make-ups were selected to be used as stimuli for the interesting task. In contrast, the uninteresting task condition used plain alphabet letters (e.g., J, Z, and Y) as stimuli. Kim et al. (2008) confirmed that the present manipulation resulted in a statistically significant difference in participants’ perceived interest in the task.

Prior to the imaging session, participants received the task instruction and practiced the task (30 trials), during which their perceived competence in performing the task was manipulated using bogus performance feedback (Sansone, 1989). The task was presented using E-Prime software. Participants assigned to the high-competence group received positive performance feedback after every five trials, in which their performance was reported to be above the norm score. The participants were informed that this norm was established through a large database. After the practice run, they were notified that their final score was 22 out of 30, with a norm of 9.3. Participants assigned to the low-competence group received negative performance feedback after every five trials that they were below the norm score. The final score shown to the low-competence group was 8 out of 30, with a norm of 18.3. A manipulation check confirmed that the high-competence group rated their perceived competence to be significantly higher (*M* = 3.31, SD = 0.79) than did the low-competence group (*M* = 2.10, SD = 0.56), *t*(23) = 4.36, *p* < 0.001.

**Figure 1:**
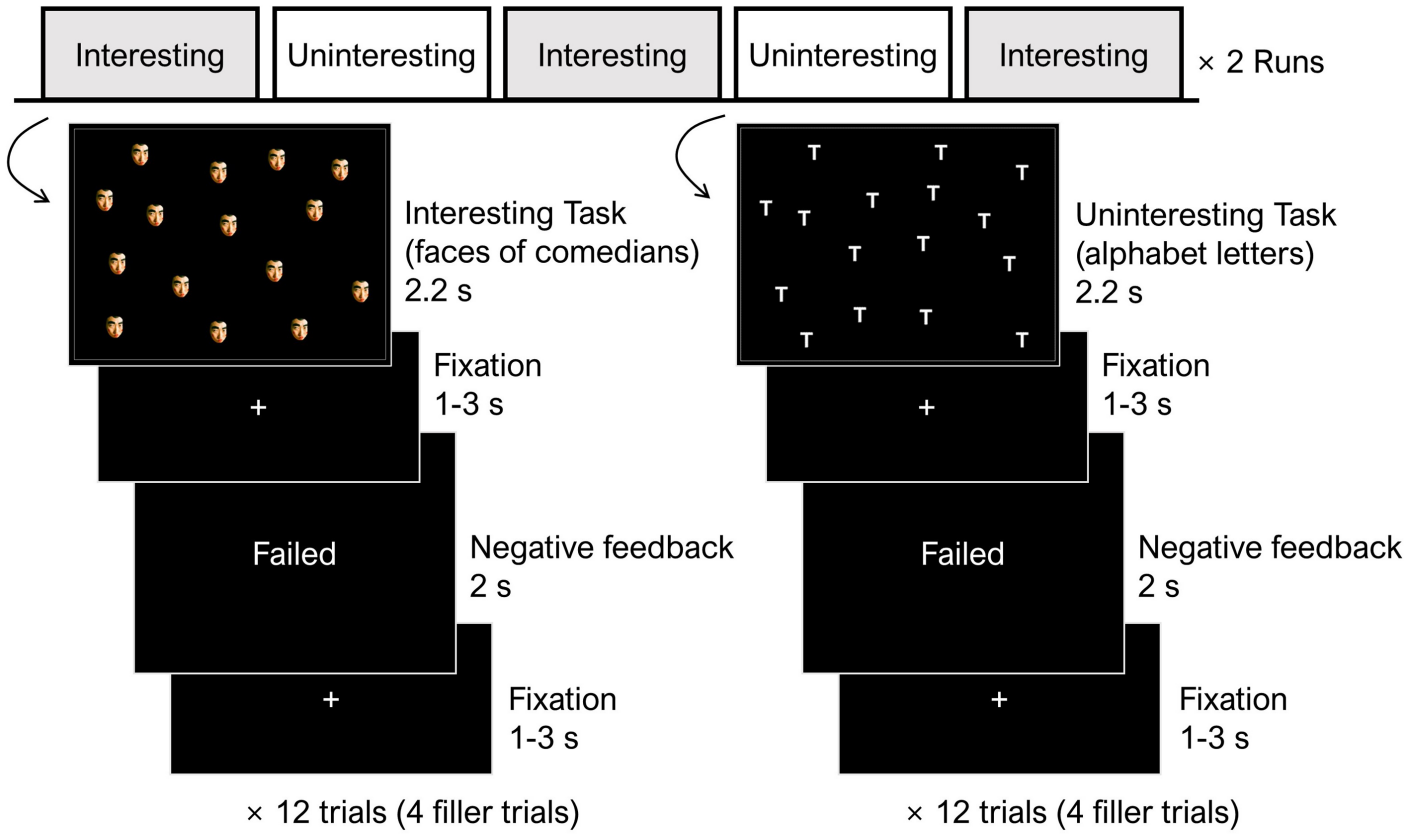
The fMRI experimental procedure of the present study. Prior to the fMRI session, participants were randomly assigned to either the high- or low-competence group for the fMRI task and were subject to the respective manipulations. Two fMRI runs, each with five blocks of 12 trials, were carried out. There were five interesting task blocks and five uninteresting task blocks, which alternated. The stimuli for the interesting task were the faces of famous comedians while those of the uninteresting task were plain alphabet letters. In each task, the participants were asked to judge whether the number of target stimuli was 15 or not as quickly as possible. Following the participants’ response, negative bogus feedback was given. To make the bogus feedback more believable, four positive feedbacks (i.e., succeeded) were included as filler trials and displayed randomly in each block.

Figure 1 presents a schematic diagram of the fMRI task. During the fMRI scan, the participants completed two runs consisting of 10 blocks, each with 12 trials. There were five interesting task blocks and five uninteresting task blocks, which were presented alternatively. In each trial, the task was displayed for 2.2 s, and participants were asked to submit their responses within this time. Following a jitter of 1–3 s, participants were presented with negative feedback (i.e., “Failed”) for 2 s. To make the negative feedback more believable, we included four positive feedback signals (i.e., “Succeeded”) per block as filler trials and interspersed them randomly. Participants were fully debriefed after completing the fMRI session.

### Data Acquisition and Analysis

All fMRI data were acquired using a 1.5 Tesla Siemens whole-body scanner with a T2^*^ weighted echo-planar imaging (EPI) sequence (TR = 2,000 ms, TE = 35 ms, flip angle = 90°, FOV = 240 mm). The acquired images were preprocessed and analyzed using SPM12 (Statistical Parametric Mapping). Specially, all data were subjected to slice-timing correction, realignment, spatial normalization, and smoothing procedures [a Gaussian filter of 8 mm full width at half maximum (FWHM)]. The participants’ preprocessed images were then analyzed using a general linear model (GLM). A total of 10 regressors was modeled for each participant. Two main regressors represented negative feedback conditions for the interesting task and for the uninteresting task. There were eight regressors of no interest, including a regressor for filler trials (positive feedback conditions for the interesting and uninteresting task), baseline fixation, and six head motion parameters to account for residual signal changes due to head movement.

To determine whether the neural responses for negative feedback processing differed according to the competence manipulation, task interest, and the interaction between the two, we contrasted brain activation patterns by group and task. In the first-level individual analysis, we created two contrast images per participant reflecting the overall effect (for the purpose of computing the main effect of group) and main effects of interesting task (interesting task [I] > uninteresting task [UI]). In the second-level random effect analysis, we performed two independent sample t-tests on the first-level contrast images to examine the main effect of group (high-competence [HC] > low-competence [LC]) and two interaction effects between group and task (HC [I-UI] > LC [I-UI], LC [I-UI] > HC [I-UI]). The main effect of interesting task was computed using a one-sample t-test. When regions showing significant interactive effects for the group and task fell within the cognitive and affective control networks, we carried out post-hoc analysis of the respective regions using the contrast estimates of the peak voxels to compare their BOLD signal changes across the group and task.

## Results

### Whole-Brain Analysis

#### Main Effects of Competence and Task Interest

We first contrasted the brain activations of negative feedback processing between two between-subject competence groups and between two within-subject task interestingness conditions. Table 1 presents the results of the main effect analysis. At a threshold of *p* < 0.005 (uncorrected), the regions exhibiting a significant main effect for group (HC > LC) included the left inferior temporal gyrus (ITG; BA 20), right superior temporal gyrus (STG; BA 22, 41), right parahippocampus (BA 35), and right middle temporal gyrus (MTG). Areas showing a significant main effect for interest (I > UI) included the left inferior frontal gyrus (IFG; BA 47), bilateral insular cortex (BA 13), right STG (BA 22, 38), bilateral middle frontal gyrus (MFG; BA 10), and right dlPFC (BA 46).

**Table 1:** Regions showing the significant main effects of group and task.

Brain regions	BA	R/L	MNI coordinates			*t*-value
			*x*	*y*	*z*	
*Group main effect (High competence > Low competence)*						
Inferior temporal gyrus	20	L	−52	−18	−24	4.29
		L	−50	−24	−18	3.28
Superior temporal gyrus	22	R	60	−4	2	3.96
Parahippocampus	35	R	20	−26	−18	3.77
Superior temporal gyrus		R	54	−20	4	3.73
Middle temporal gyrus	21	R	50	−10	−20	3.69
Superior temporal gyrus	41	R	48	−30	6	3.49
*Task main effect (Interesting task > Uninteresting task)*						
Inferior frontal gyrus, Insular cortex	47	L	−34	26	−4	4.68
Occipital lobe		R	32	−72	0	4.18
Inferior parietal lobule		R	60	−32	34	3.89
Postcentral gyrus	2, 3	R	44	−26	42	3.86
Superior temporal gyrus	22	R	50	−8	0	3.84
Superior temporal gyrus	38	R	52	16	−10	3.77
Middle frontal gyrus		R	38	36	30	3.67
Hippocampus		R	32	−36	−6	3.65
Middle frontal gyrus	10	L	−42	54	−2	3.64
Insular cortex	13	R	34	22	16	3.56
Insular cortex	13	R	34	−32	14	3.54
Middle frontal gyrus, dlPFC	10, 46	R	34	48	10	3.45
		R	44	48	12	3.34
Angular gyrus		L	−48	−58	36	3.39

*All regions are significant at p < 0.005, uncorrected with an extent threshold of 10 voxels. BA, Brodmann’s area; R/L, Right or left hemisphere; and dlPFC, dorsolateral prefrontal cortex*.

#### Interaction Effect of Competence and Task Interest

We then examined whether there were significant differences in the cognitive and affective control network responsivity by the group and task. Table 2 shows the results of the interaction effects for the group and task. As hypothesized, activities in the cognitive control network were found to be greater in the high-competence group than in the low-competence group during negative feedback processing for the interesting task relative to the uninteresting task (HC [I-UI] > LC [I-UI]). These regions included the bilateral dlPFC (BA 9, 46), bilateral ACC (BA 23, 24), right inferior PFC, and right dorsal striatum. In contrast, the low-competence group exhibited stronger activation in the left thalamus after receiving negative feedback for the interesting task compared with the uninteresting task than did the high-competence group (LC [I-UI] > HC [I-UI]). There was no evidence that the amygdala response differed by group and task.

**Table 2:** Regions showing significant interaction effects of group and task.

Brain regions	BA	R/L	MNI coordinates			*t*-value	
			*x*	*y*	*z*		
*High competence (I-UI) > Low competence (I-UI)*							
dlPFC	46	R	46	44	14	4.96	
Anterior cingulate cortex	23, 24, 32, 33	R	10	−2	32	4.95	
		R	2	−8	32	4.80	
		L	−14	16	28	4.56	
Occipital lobe, Fusiform gyrus	19, 37	R	40	−64	2	4.31	
		R	22	−68	−2	4.04	
		R	30	−70	0	3.51	
Occipital lobe, Fusiform gyrus	19	L	−24	−70	−4	4.27	
Postcentral gyrus	2	L	−36	−34	46	4.27	
Occipital lobe	18	R	8	−80	20	4.09	
Postcentral gyrus	2, 3	R	38	−26	38	4.04	
Inferior prefrontal cortex		R	34	−8	34	3.97	
Dorsal striatum		R	24	8	26	3.77	
		R	32	12	30	3.16	
Anterior cingulate cortex		L	−14	−20	34	3.73	
*Low competence (I-UI) > High competence (I-UI)*							
Thalamus		L	−16	−32	4	4.11	

*All regions are significant at p < 0.005, uncorrected with an extent threshold of 15 voxels. BA, Brodmann’s area; R/L, Right or left hemisphere; I, Interesting task; UI, Uninteresting task; and dlPFC, Dorsolateral prefrontal cortex*.

### BOLD Signal Changes in Cognitive and Affective Control Areas

The interaction effects of group and task suggested differential brain activations within the networks of cognitive control (i.e., dlPFC, ACC, inferior PFC, and dorsal striatum) and negative affect (i.e., thalamus) across the group and task. To approximate the BOLD signal changes in these brain regions, we conducted post-hoc analysis of the contrast estimates with a 90% confidence interval in the peak voxels.

**Figure 2:**
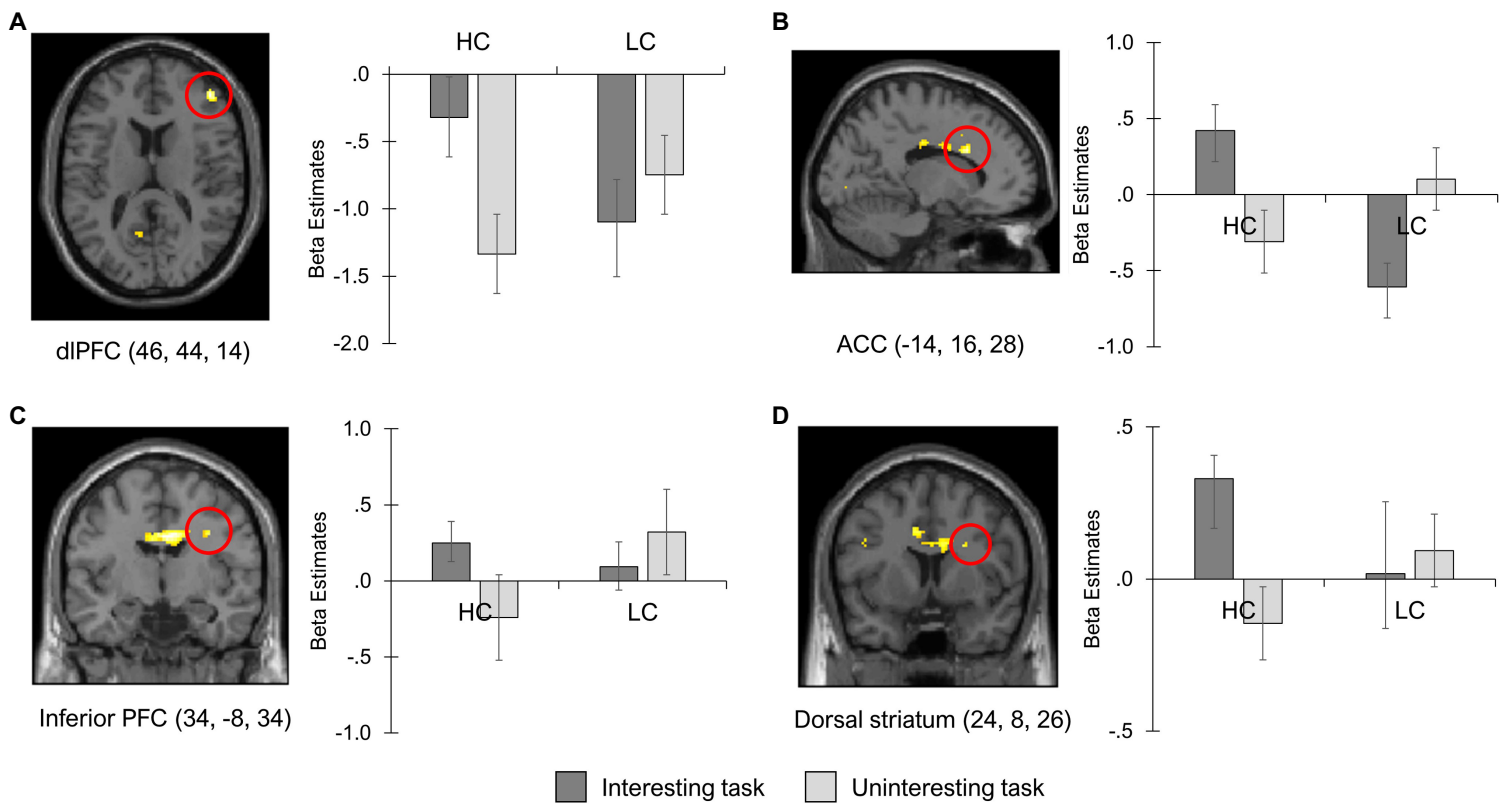
Regions showing reduced cognitive control during negative feedback processing for interesting task in low-competence group. The contrast estimates in the peak voxel of **(A)** dlPFC, **(B)** ACC, **(C)** inferior PFC, and **(D)** dorsal striatum, which showed significant interaction effects of group and task. HC, High-competence group; LC, Low-competence group; dlPFC, dorsolateral prefrontal cortex; ACC, anterior cingulate cortex; and PFC, prefrontal cortex.

Figure 2 presents the activation patterns of cognitive control regions that exhibited significant group × task effects, including the right dlPFC [MNI peak coordination (46, 44, 14)], left ACC (−14, 16, 28), right inferior PFC (34, −8, 34), and right dorsal striatum (24, 8, 26). The strongest activity in the cognitive control brain regions was observed in the high-competence individuals when they were coping with negative feedback given to the interesting task (HC-I condition). When the high-competence group received negative feedback for the uninteresting task (HC-UI condition), the responses in these areas were lower. The opposite was true for the low-competence group. The low-competence group showed greater activation in the cognitive control network during negative feedback processing of uninteresting task (LC-UI condition) rather than interesting task (LC-I condition). The cognitive control brain regions exhibited the lowest level of activation in the LC-I condition.

**Figure 3:**
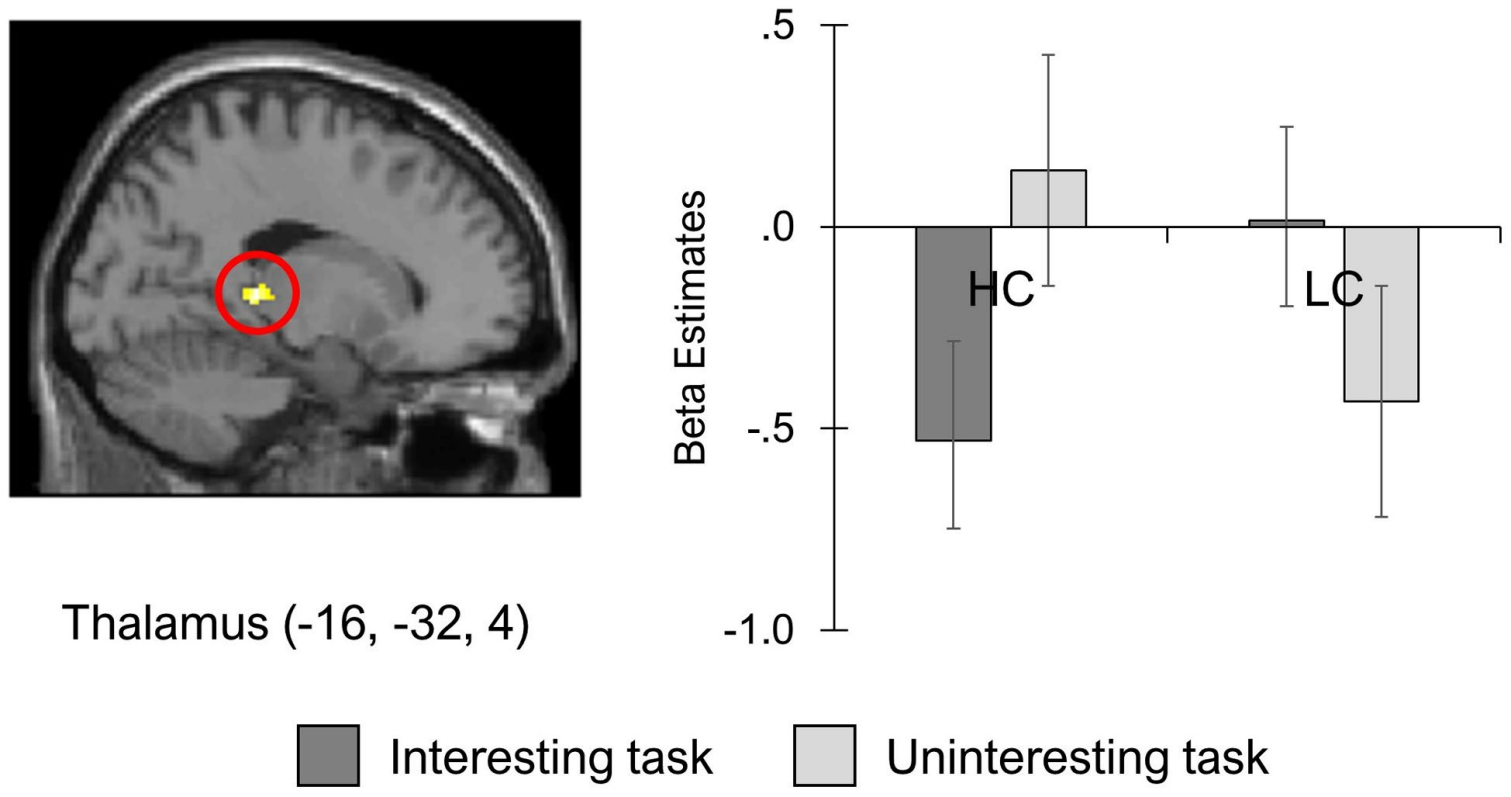
Less attenuated affective control during negative feedback processing for interesting task in low-competence group. HC, High-competence group; LC, Low-competence group.

The contrast estimate in the peak voxel of the left thalamus [MNI peak coordination (−16, −32, 4)] was also shown to produce different responses by group and task. As shown in Figure 3, the HC-I and LC-UI conditions demonstrated greater attenuation of thalamic activity. Less of a reduction in thalamus activity was observed in the HC-UI and LC-I conditions. This finding aligns with those of Figure 2 and implies that the low-competence individuals may undergo difficulty in regulating negative feedback both cognitively and emotionally when the task was interesting.

## Discussion

We examined the neural responses of high-competence and low-competence groups to negative feedback after the completion of interesting and uninteresting tasks. Distinct regions of the brain were activated when processing the same negative feedback depending on whether the participants found the task interesting and whether they were led to believe that they were competent in performing the task. The differential brain activation observed in the present study provides unique evidence that students engage in discrete cognitive and affective processes contingent upon both the levels of perceived competence and task interest when coping with failure.

### Distinct Roles of Competence and Interest in Negative Feedback Processing

The main effect analysis suggests that perceived competence and task interest uniquely recruited a discrete set of brain networks in the processing of negative feedback. High competence appears to activate brain regions responsible for regulating negative emotion and learning, such as the temporal gyrus and parahippocampus, following failure feedback. In a prior meta-analysis, Kohn et al. (2014) contended that the STG receives signals from the vmPFC when there is a need for the regulation of negative emotion and execute the regulation process. The activation of the parahippocampus, an area related to learning and memory (Degonda et al., 2005), suggests the utilization of memory resources when processing negative feedback. This could mean that highly competent participants actively encode the current feedback and retrieve previous feedback to monitor and rectify their performance in the face of failure (Kim et al., 2010). Therefore, high competence may aid students in managing failure by controlling their negative emotion and allocating greater memory resources to improving future performance.

When given negative feedback on an interesting task, distributed brain areas associated with emotion regulation and cognitive control were also activated. Analogous to the results for high competence, the main effect of interest produced significant responses in the STG and hippocampus, possibly indicating processes aimed at regulating negative emotion and learning. High interest also activated the IFG, MFG, and angular gyrus, all known to serve pivotal roles in overcoming negative emotion (Grecucci et al., 2013; Kohn et al., 2014). Other regions uniquely recruited by the interesting task included the insular cortex and dlPFC, which constitute the core regions of the cognitive control network (Miller and Cohen, 2001; Cole and Schneider, 2007). Taken together, task interest seems to increase signals in the areas responsible for the experience of negative affect and cognitive effort to cope with these feelings.

### Joint Effects of Competence and Interest on Negative Feedback Processing

Despite these significant main effects, the results of the interaction effect and ROI analysis suggested that the modulation of the cognitive control and negative affect areas when given negative feedback depended on both perceived competence and task interest. As predicted, parts of the cognitive control network, such as the dlPFC, ACC, inferior PFC, and dorsal striatum, were activated in the high-competence group when they received negative feedback on the interesting task. Activity in the thalamus was attenuated for the same condition. When high-competence individuals received negative feedback after completing the uninteresting task, the activity in the cognitive control network was suppressed, and that in the thalamus was less attenuated. The opposite was true for individuals with low competence. Whereas the brain regions associated with cognitive control were activated in the low-competence group when they received negative feedback on the uninteresting task, the activity in these areas was reduced for the interesting task. Consistent with our hypothesis, the activation in the thalamus was less attenuated during the interesting task than the uninteresting one in the low-competence group. These findings suggest that individuals with high perceived competence are better at cognitive control and emotion regulation in response to negative performance feedback following an interesting task relative to an uninteresting one. In contrast, individuals with low perceived competence could do so when they find the task uninteresting rather than interesting.

Our findings align closely with the assertions of the EVT, lending a robust neuroscientific basis for the theory. The multiplicative patterns are upheld even when students are confronted with the negative consequences of a task. More importantly, our results suggest the possible mechanisms underlying the discrepancy between perceived competence and task interest. The activity in the thalamus was less attenuated in the HC-LI and LC-HI conditions, which may be indicative of perceived threat and increased anxiety (Choi et al., 2012). The deficiency in cognitive control when challenged may be another mechanism. Higher activation of the cognitive control network was observed in the HC-I and LC-UI conditions, while the activity in these regions was much lower in the HC-UI and LC-I conditions. Future studies should extend these findings and investigate the failure in cognitive control as a potential explanation for maladaptive tendencies when either competence or interest falls behind.

Another plausible explanation for the observed interaction effects is the idea of seductive detail. While the positive effects of interest on learning have been well documented, many have also pointed out that it can be a source of disruption. The widely accepted notion of seductive detail effect assumes that interesting but tangential sources (e.g., funny images and interesting stories) can distract students from attending to key concepts, impeding their learning (Harp and Mayer, 1998; for a review, see Rey, 2012). More recently, Senko et al. (2021) showed that interesting materials could also inflate students’ judgment of learning and lead them to overestimate their performance. That is, more interesting materials can lead to a flawed conception of the student’s mastery of the task. Students with low competence may be especially vulnerable to this trap (Sanchez and Wiley, 2006; Alexander, 2019). This is because students with high competence have the capacity and willingness to process materials at a deeper level and distinguish between relevant and irrelevant contents, while those with low competence may lack this ability (Alexander and Jetton, 1996; Alexander, 2016). As such, negative feedback following an interesting task may be more painful for low-competence students due to the false conception of task mastery, hampering their ability to control the situation cognitively and emotionally.

### Implications and Limitations

Our findings extend the understanding of the varying mechanisms associated with negative feedback processing according to task and individual characteristics and suggest adaptive instruction practices tailored for different learners. From an empirical perspective, the present results advance both the literature on negative feedback processing and motivation by identifying preferential neural substrates potentially associated with failure by perceived competence and task interest. Our findings indicate that it would be important to consider task and individual characteristics together, rather than each factor in isolation, in examining negative feedback processing.

From a pragmatic perspective, this study offers recommendations for designing an adaptive classroom environment. First, teachers need to be cautious when providing interesting materials to students who lack competence in performing a task because this type of material could have unfavorable outcomes if they fail. Nonetheless, this does not necessarily mean that interesting materials should be avoided for these students. Instead, for students with low-competence beliefs, simple and rudimentary materials that can provide them an opportunity to acquire, practice, and build basic skills and competency can be used before moving on to more interesting tasks. For students with high-competence beliefs, interesting materials can act as a buffer against negative feedback. Hence, triggering students’ interest with novel or humorous stimuli can help them to endure failure. Alternatively, teachers can offer highly competent students a chance to discover or develop their own interest in the materials, for instance, by asking them to think about the personal value of the material (e.g., Hulleman and Harackiewicz, 2009). Ultimately, these instructional practices would help students better identify errors, learn from their mistakes, and improve their performance even when experiencing temporary failure by engaging in various cognitive and affective control processes.

The limitations of this study should also be acknowledged. The study’s major limitation was the artificial nature of the task and feedback. Because we provided failure feedback for a novel and relatively simple experimental task, both the authenticity and intensity of the feedback may have been weaker than what students might experience in reality. For example, in a typical classroom, where negative feedback prevails and has greater personal significance, much stronger emotional reactions can be expected. These responses could be differently affected by task interest and competence beliefs. To test this possibility, future studies should examine the interaction effects of competence and interest for a task that students place a greater value and importance (e.g., academic subject and intelligence test).

In addition, the facts that we did not check participants’ interest in the task directly and used rather a lenient threshold in reporting the neuroimaging results need to be improved in future studies. Overall, it is important to continue investigating the feedback effects on the brain of adolescents in learning contexts. This is because, although failure is unavoidable in most learning situations, student motivation, emotion, and behavior are strongly swayed by it. Thus, future work should extend the present findings and examine effective ways to use feedback that can minimize the potentially debilitating impact on students by looking at the types (e.g., informative and confirmatory), frequency, and different consequences (e.g., motivation, emotion, and behavior) of negative feedback using brain imaging. Investigating these factors promises to yield more profound insights into feedback processing, which can help to nurture successful learners.

## Data Availability Statement

The raw data supporting the conclusions of this article will be made available by the authors, without undue reservation.

## Ethics Statement

The studies involving human participants were reviewed and approved by Korea University. The patients/participants provided their written informed consent to participate in this study.

## Author Contributions

All authors have made a substantial, direct, and intellectual contribution and agreed to be accountable for the content presented in this work.

## Funding

This research was supported by the College of Education, Korea University Grant in 2020.

## Conflict of Interest

The authors declare that the research was conducted in the absence of any commercial or financial relationships that could be construed as a potential conflict of interest.

## Publisher’s Note

All claims expressed in this article are solely those of the authors and do not necessarily represent those of their affiliated organizations, or those of the publisher, the editors and the reviewers. Any product that may be evaluated in this article, or claim that may be made by its manufacturer, is not guaranteed or endorsed by the publisher.
